# Outcomes of uninterrupted vs interrupted Periprocedural direct oral Anticoagulants in atrial Fibrillation ablation: A meta‐analysis

**DOI:** 10.1002/joa3.12507

**Published:** 2021-01-29

**Authors:** Indranill Basu‐Ray, Dibbendhu Khanra, Péter Kupó, Jared Bunch, Sue A. Theus, Anindya Mukherjee, Sumit K. Shah, András Komócsi, Adedayo Adeboye, John Jefferies

**Affiliations:** ^1^ Department of Cardiology Memphis VA Medical Center Memphis TN USA; ^2^ The University of Tennessee Health Science Center Memphis TN USA; ^3^ All India Institute of Medical Sciences Rishikesh India; ^4^ New Cross Hospital Royal Wolverhampton NHS Trust Wolverhampton UK; ^5^ University of Szeged Szeged Hungary; ^6^ Intermountain Heart Institute Intermountain Medical Center Murray UT USA; ^7^ Memphis VA Medical Center Memphis TN USA; ^8^ Department of Cardiology NRS Medical College Kolkata India; ^9^ University of Arkansas for Medical Sciences Little Rock AR USA; ^10^ Department of Cardiology University of Pécs Pécs Hungary

**Keywords:** atrial fibrillation, bleeding, catheter ablation, direct oral anticoagulants, silent stroke

## Abstract

**Background:**

Studies indicate that uninterrupted anticoagulation (UA) is superior to interrupted anticoagulation (IA) in the periprocedural period during catheter ablation of atrial fibrillation. Still IA is followed in many centers considering the bleeding risk. This meta‐analysis compares interrupted and uninterrupted direct oral anticoagulation during catheter ablation of atrial fibrillation.

**Methods:**

A systematic search into PubMed, EMBASE, and the Cochrane databases was performed and five studies were selected that directly compared IA vs UA before ablation and reported procedural outcomes, embolic, and bleeding events. The primary outcome of the study was major adverse cerebro‐cardiovascular events.

**Results:**

The meta‐analysis included 840 patients with UA and 938 patients with IA. Median follow‐up was 30 days. Activated clotting time (ACT) before first heparin bolus was significantly longer with UA (*P *= .006), whereas mean ACT was similar between the two groups (*P *= .19). Total heparin dose needed was significantly higher with IA (mean, ‒1.61; 95% CI, ‒2.67 to ‒0.55; *P *= .003). Mean procedure time did not vary between groups (*P *= .81). Overall complication rates were low, with similar major adverse cerebro‐cardiovascular event (*P *= .40) and total bleeding (*P *= .55) rates between groups. Silent cerebral events (SCEs) were significantly more frequent with IA (log odds ratio, ‒0.90; 95% CI, ‒1.59 to ‒0.22; *P *< .01; *I* ^2^, 33%). Rates of major bleeding, minor bleeding, pericardial effusion, cardiac tamponade, and puncture complications were similar between groups.

**Conclusions:**

UA during atrial fibrillation ablation has similar bleeding event rates, procedural times, and mean ACTs as IA, with fewer SCEs.

## Graphical abstract

This meta‐analysis compares interrupted and uninterrupted direct oral anticoagulation during catheter ablation of atrial fibrillation. Uninterrupted anticoagulation during atrial fibrillation ablation has similar bleeding event rates, procedural times, and mean activated clotting times as interrupted anticoagulation, with fewer silent cerebral events.

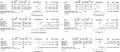

## 1 INTRODUCTION

Catheter ablation of atrial fibrillation (AF) has expanded enormously over recent years, given improvements in available hardware, newer technologies, and growing evidence that the procedure is effective for rhythm control in patients with AF.^1^ Although catheter ablation of AF is relatively safe in experienced hands, it is occasionally complicated by periprocedural thromboembolism, including stroke or transient ischemic attack (TIA), resulting from catheter manipulation and lesion creation in the left atrium; further, puncture complications and cardiac tamponade are not uncommon, because of multiple large sheaths and background anticoagulation.^2^ Understandably, determining the optimum anticoagulation regimen for catheter ablation of AF is of utmost importance, both to balance the risks for ischemic and bleeding events during the procedure and to accommodate same‐day discharge protocols.^3^

Direct oral anticoagulants (DOACs), including dabigatran, rivaroxaban, apixaban, and edoxaban, have largely replaced the vitamin K antagonist warfarin in recent years, as they are associated with lower risk for bleeding events and thus better stroke prevention in patients with AF.^4^ Even so, many operators believe it wise to allow a 24‐hour gap in the DOAC regimen before catheter ablation of AF to avoid bleeding risks, despite the fact that guidelines recommend uninterrupted DOAC administration in the periprocedural period^5^, ^6^, ^7^ and that studies have shown better results from uninterrupted vs interrupted anticoagulation regimens, with better prevention of embolic events.^8^ Studies addressing the safety and efficacy of an interrupted DOAC regimen during catheter ablation of AF are few and are limited by small sample sizes, short follow‐up periods, rare events, and variable outcomes. We therefore conducted a meta‐analysis comparing procedural characteristics and embolic and bleeding events between uninterrupted and interrupted DOAC regimens for catheter ablation of AF.^9^

## 2 METHODS

### 2.1 Search strategy

A systematic review was performed to search the existing literature as of April 2020. Three physician reviewers (DK, AM, and SS) queried PubMed, EMBASE, and the Cochrane Central Register of Controlled Trials (CENTRAL) databases for published literature; search terms were “atrial fibrillation,” “catheter ablation,” “radiofrequency ablation,” “cryoballoon,” “hot balloon,” “uninterrupted,” “interrupted,” “novel oral anticoagulants,” “direct oral anticoagulants,” “dabigatran,” “rivaroxaban,” “apixaban,” “edoxaban,” “stroke,” “silent cerebral events,” and combinations of these keywords. Additional literature was sought by searching the references of eligible articles. Any inter‐reviewer discrepancies were resolved by a fourth reviewer (IBR).

### 2.2 Study selection

For the qualitative synthesis of the meta‐analysis, we selected studies that (a) directly compared uninterrupted anticoagulation (UA) vs interrupted anticoagulation (IA) with a DOAC regimen before catheter ablation of AF and (b) provided procedural outcomes and embolic and bleeding events. Studies that involved both UA and IA with DOACs but did not report comparative outcome data for each regimen were excluded from the quantitative meta‐analysis. Single‐arm studies, case reports, case series, and cohort studies that had < 10 participants or that did not present adequate safety or efficacy outcome data also were excluded. See eFigure 1 in the Online Supplement.

### 2.3 Data extraction

Baseline characteristics and safety and efficacy outcome data were extracted from each of the selected studies and entered into a Microsoft Excel spreadsheet by authors DK, AM, and SS. Baseline characteristics included DOAC regimen, number of participants, maximum follow‐up duration, age, sex, CHA_2_DS_2_‐VASc (congestive heart failure, hypertension, age ≥ 75 years, diabetes mellitus, stroke or TIA, vascular disease, age 65 to 74 years, and sex category) score, HAS‐BLED (hypertension, abnormal renal or liver function, stroke, bleeding, labile international normalized ratio, elderly, drugs, or alcohol) score, left ventricular ejection fraction (LVEF), left atrium diameter, creatinine clearance, associated antiplatelet drugs, dimerized plasmin fragment D (D‐dimer) and brain natriuretic peptide levels, and presence of paroxysmal AF, coronary artery disease, chronic kidney disease, or structural heart disease. Procedural outcomes included procedure time, activated clotting time (ACT), heparin dose, cardioversion, and use of protamine. Efficacy outcomes included embolic events and silent cerebral events (SCEs). Safety outcomes included major bleeding events (eg, cardiac tamponade, pseudoaneurysm, retroperitoneal hematoma, and intracranial hemorrhage) and minor bleeding events (eg, groin hematoma, pericardial effusions, and rebleeding from venous sites).

### 2.4 Outcomes

The primary outcome of the study was major adverse cerebro‐cardiovascular events (MACCVEs), which was a composite of stroke or TIA and major bleeding, total bleeding (composite of major and minor bleedings), and SCE. The secondary outcomes were cerebral embolic stroke or TIA, major and minor bleeding, total pericardial effusion, cardiac tamponade, and total puncture complications (composite of pseudoaneurysms, retroperitoneal hematomas, and rebleeding from venous sites).

### 2.5 Data analysis

To compare the safety and efficacy outcomes in the UA and IA groups, we used hypergeometric‐normal modeling to approximate the exact likelihood, as the number of events in each study was small relative to group size and included many zero events. To negate the small study effect, we calculated logarithmic odds ratios (log ORs) with 95% CIs and then used R software^10^ to back‐transform the results to predicted exponential ORs and 95% CIs.^11^ Heterogeneity was assessed by I^2^, and publication bias was assessed by funnel plot.

## 3 RESULTS

Five studies with a total of 840 UA patients and 938 IA patients were included in the meta‐analysis; of these, three were randomized trials,^12^, ^13^, ^14^ and two were observational studies.^15^, ^16^ Two identified studies were excluded because of lack of comparative data.^17^, ^18^ See eFigure 1 in the Supplement. The three randomized studies were critically appraised using the Risk of Bias 2.0 Scale, and the two observational studies were appraised using the Newcastle‐Ottawa Scale (eTable 1 in the Supplement).

### 3.1 Baseline characteristics

The various anticoagulant regimens are described in Table 1, along with baseline characteristics across the five studies. Follow‐up periods differed across studies; the median duration being 30 days. Mean age, mean CHA_2_DS_2_‐VASc score, and the number of participants who had paroxysmal AF, had received antiplatelet drugs, or had structural heart disease were similar in both UA and IA groups across all studies. Maximum left atrial diameter, LVEF, creatinine clearance, and D‐dimer and brain natriuretic peptide levels did not vary significantly between the UA and IA groups.

**TABLE 1:** Descriptive comparison of baseline characteristics^a^

	Randomized studies						Observational studies			
	Reynolds et al (2018)^12^		Nagao et al (2019)^13^		Nakamura et al (2019)^14^		Müller et al (2016)^15^		Nakamura et al (2019)^16^	
	UA (n = 150)	IA (n = 145)	UA (n = 100)	IA (n = 100)	UA (n = 421)	IA (n = 423)	UA (n = 64)	IA (n = 42)	UA (n = 105)	IA (n = 228)
DOAC	A5 150 (100) A2.5 0 (0)	A5 113 (98) A2.5 32 (2)	R/E 49 (49) A 51 (51) LD 48 (48)	R/E 53 (53) A 47 (47) LD 41 (41)	D 27 (6) R 160 (38) A 117 (28) E 117 (28)	D 38 (9) R 151 (36) A 125 (30) E 109 (26)	—	—	D 28(26) R 34 (32) A 10 (9) E 7(6)	D 43(18) R 88 (38) A 38 (16) E 59(25)
Last dose taken on	Same day Usual dose	Last day Usual dose	Same day Usual dose	Last day Usual dose	Same day Full doses UFH 24 h	Last day Full dose UFH 24 h	Same day Half doses	24 h before Bridging	Same day Full doses	Last day Full dose
Resumed on	Next dose	Next dose	Next dose	Next dose	Next dose	Next day	Next dose	Next day	Next dose	Next morning
Maximum follow‐up, days	30	30	30	30	30	30	30	30	1	1
Age, y	62.8 ± 9.9	64.3 ± 10.3	70 ± 29	70 ± 28	65 ± 10	65 ± 10	63.5 ± 1.2	62.8 ± 1.5	6 4.4 ± 10.9	6 4.3 ± 11.6
Female	49 (33)	48 (33)	36 (36)	38 (38)	123 (29)	125 (93)	24 (37.5)	14 (33)	36 (35)	75 (33)
CHA_2_DS_2_‐VASc score	2.2 ± 1.6	2.4 ± 1.6	2.8 ± 1.6	2.6 ± 1.5	—	—	2.3 ± 0.1	2.4 ± 0.2	1.9 ± 1.4	1.9 ± 1.4
HAS‐BLED score	1.0 ± 0.9	1.1 ± 0.8	—	—	—	—	—	—	—	—
Paroxysmal AF	100 (67)	91 (63)	57 (57)	59 (59)	222 (53)	236 (56)	28 (44)	19 (45)	—	—
LVEF, %	56.0 ± 9.2	57.3 ± 8.1	66 ± 9	65 ± 10	61 ± 11	61 ± 9	56.6 ± 1.0	58.9 ± 0.8	66.5 ± 8.4	66.4 ± 8.2
Maximum LAD, mm	—	—	40 ± 6	40 ± 6	41 ± 7	41 ± 7	44.2 ± 0.8	42.9 ± 0.8	39.2 ± 6	39 ± 6.1
CAD	42 (28)	25 (17)	12 (12)	9 (9)	—	—	—	—	6 (5)	18(7)
CKD	5 (3)	8 (6)	63 (63)	50 (50)	—	—	—	—	—	—
Creatinine clearance, mg/mL	—	—	79 ± 35	81 ± 29	80.0 ± 26.3	80.0 ± 27.3	—	—	57. 4 ± 26.5	56.9 ± 25.8
Structural heart disease	16 (10)	143 (10)	‒	‒	49 (12)	60 (14)	—	—	‐	‐
Antiplatelets	43 (29)	28 (19)	27 (27)	31 (31)	—	—	—	—	7 (6)	17 (7)
D‐dimer, μg/mL	—	—	0.7 ± 0.4	0.7 ± 0.6	0.4 ± 0.6	0.5 ± 0.3	—	—	—	—
BNP, pg/mL	—	—	141 ± 333	124 ± 165	108.3 ± 148.1	101.9 ± 123.1	—	—	—	—

Note

NB all patients in Nakamura's observational study^16^ had paroxysmal AF.

Abbreviations: A, apixaban; AF, atrial fibrillation; BNP, brain natriuretic peptide; CAD, coronary artery disease; CHA_2_DS_2_‐VASc, congestive heart failure, hypertension, age ≥ 75 years, diabetes mellitus, stroke or transient ischemic attack, vascular disease, age 65 to 74 years, sex category; CKD, chronic kidney disease; D, dabigatran; D‐dimer, dimerized plasmin fragment D; DOAC, direct oral anticoagulant; E, edoxaban; HAS‐BLED, hypertension, abnormal renal or liver function, stroke, bleeding, labile international normalized ratio, elderly, drugs or alcohol; IA, interrupted anticoagulation group; LAD, left atrial diameter ;LD, low dose; LVEF, left ventricular ejection fraction; R, rivaroxaban; UA, uninterrupted anticoagulation group; UFH, unfractionated heparin.

^a^ Values are shown as n (%) or mean ± SD.

### 3.2 Procedural data

**FIGURE 1:**
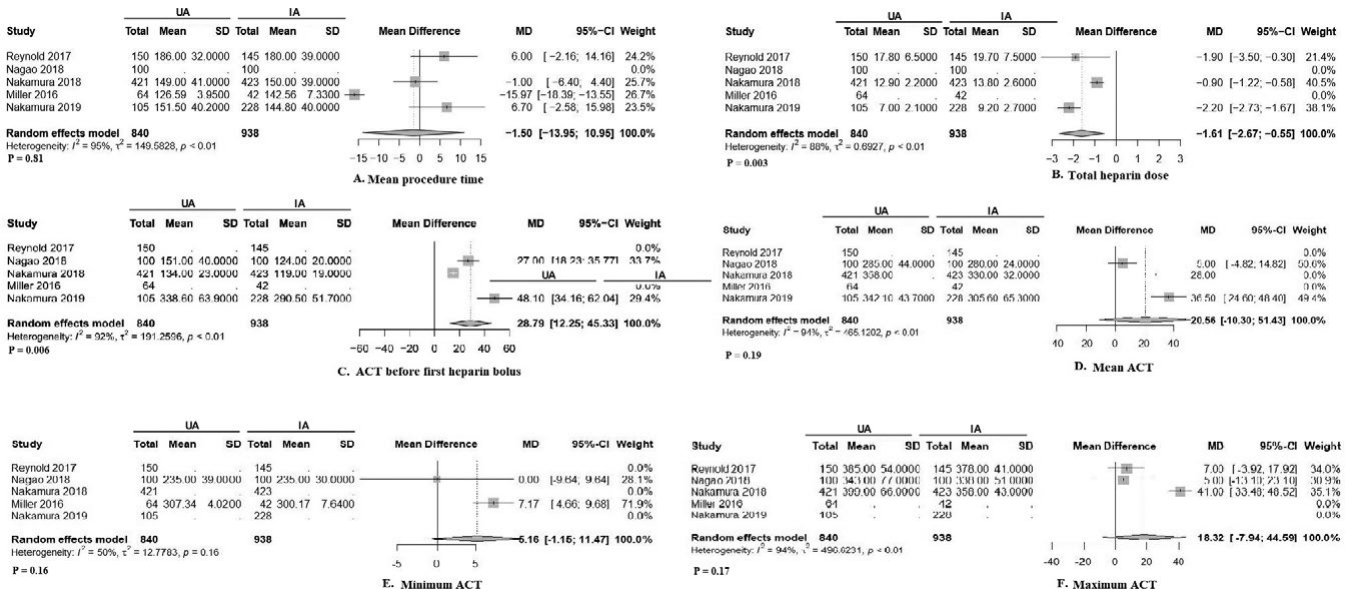
Statistical comparison of procedural characteristics between uninterrupted and interrupted direct oral anticoagulation in patients undergoing catheter ablation of atrial fibrillation. Abbreviations: ACT, activated clotting time; IA, interrupted anticoagulation group; MD, mean difference; UA, uninterrupted anticoagulation group

Figure 1 and Table 2 show statistical comparisons of procedural characteristics between the UA and IA groups in patients undergoing catheter ablation of AF. Total heparin dose needed was significantly higher in the IA group (mean, ‒1.61; 95% CI, ‒2.67 to ‒0.55; *P *= .003; *I* ^2^, 88%). ACT before first heparin bolus was significantly longer in the UA group (mean, 28.79; 95% CI, 12.25 to 45.33; *P *= .006; *I* ^2^, 92%). No significant differences between the UA and IA groups were found for mean procedure time (mean, ‒1.50; 95% CI, ‒13.95 to 10.95; *P *= .81; *I* ^2^, 95%), mean ACT (mean, 20.56; 95% CI, ‒10.30 to 51.43; *P *= .19; *I* ^2^, 94%), maximum ACT (mean, 18.32; 95% CI, ‒7.94 to 44.59; *P *= .17; *I* ^2^, 94%), or minimum ACT (mean, 5.16; 95% CI, ‒1.15 to 11.47; *P *= .16; *I* ^2^, 50%). Protamine use was marginally higher in the UA group, but the difference not statistically significant (OR, 2.53; 95% CI, 1.59 to 4.00; *P *= .06; *I* ^2^, 73%), as shown in eFigure 2 in the Supplement.

**TABLE 2:** Comparison of procedural details^a^

	Randomized studies						Observational studies			
	Reynolds et al (2018)^12^		Nagao et al (2019)^13^		Nakamura et al (2019)^14^		Müller et al (2016)^15^		Nakamura et al (2019)^16^	
	UA (n = 150)	IA (n = 145)	UA (n = 100)	IA (n = 100)	UA (n = 421)	IA (n = 423)	UA (n = 64)	IA (n = 42)	UA (n = 105)	IA (n = 228)
Morning session	53 (53)	58 (58)	53 (53)	58 (58)	169 (40)	166 (39)	—	—	—	—
Balloon‐assisted ablation	46 (31)	38 (26)	—	—	39 (9)	28 (7)	—	—	105 (100)	228 (100)
Adjunctive ablation lesion	73 (49)	77 (53)	—	—	44 (11)	49 (12)	—	—	22 (20)	55 (24)
Double transseptal puncture	80 (54)	83 (58)	—	—	—	—	—	—		
Procedure time, min	186 ± 32	180 ± 39	—	—	149 ± 41	150 ± 39	126.6 ± 4.0	142.6 ± 7.3	151.5 ± 40.2	144.8 ± 40.0
LA dwelling time, min	—	—	—	—	—	—	—	—	76.3 ± 33.9	80.2 ± 35.3
Application time, min	29 ± 12	31 ± 13	—	—	—	—	—	—	—	—
Fluoroscopy time, min	—	—	—	—	—	—	20.7 ± 0.9	21.2 ± 1.1	‒	‒
ACT before heparin bolus, s	—	—	151 ± 40^b^	124 ± 20^b^	134 ± 23 ^b^	119 ± 19^b^	—	—	338.6 ± 63.9^b^	290.5 ± 51.7^b^
Total heparin dose, 10^3^ units	17.8 ± 6.5^b^	19.7 ± 7.5^b^	136 ± 60(U/kg)	153 ± 61 (U/kg)	12.9 ± 2.2 ^b^	13.8 ± 2.6^b^	—	—	7. 0 ± 2.1^b^	9.2 ± 2.7^b^
Maximum ACT, s	385 ± 54	378 ± 41	343 ± 77	338 ± 51	399 ± 66	358 ± 43^b^	—	—	—	—
Minimum ACT, s	—	—	235 ± 39	235 ± 30	—	—	307.3 ± 4.0	300.2 ± 7.6	—	—
Mean ACT, s	—	—	285 ± 44	280 ± 24	358 ± 49	330 ± 32^b^	—	—	342.1 ± 43.7^b^	305.6 ± 65.3^b^
Protamine given	137 (91)	128 (88)	—	—	405 (96) ^b^	371 (88)^b^	—	—	—	—
Protamine dose, mg	56.1 ± 25.8	56.9 ± 24.8	—	—	—	—	—	—	—	—
Cardioversion	—	—	35 (35)	33 (33)	—	—	69 (46)	16 (39)	37 (35)	77 (33)

Abbreviations: ACT, activated clotting time; IA, interrupted anticoagulation group; LA, left atrial; UA, uninterrupted anticoagulation group.

^a^ Values shown are n (%) or mean ± SD.

^b^ Denoting a significant difference between groups.

### 3.3 Outcomes

Clinical outcomes across the studies are described in eTable 2 in the Supplement, and statistical comparisons of these outcome characteristics between the UA and IA groups are outlined in Table 3.

**TABLE 3:** Statistical comparison of outcome characteristics between uninterrupted and interrupted direct oral anticoagulation in patients undergoing catheter ablation of atrial fibrillation

	UA (e/n)	IA (e/n)	Log OR (95% CI)	*P* value	*Z* value	*I* ^2^ (%)	Tau^2^	Predicted OR (95% CI)
Primary outcomes								
MACCVE	7/840	12/938	‒0.40 (‒1.33 to 0.53)	.40	‒0.85	0	0	0.67 (0.26 to 1.70)
Total bleeding	54/735	58/710	‒0.12 (‒0.51 to 0.27)	.55	‒0.60	0	0	0.89 (0.60 to 1.31)
Silent cerebral events	95/617	169/683	‒0.90 (‒1.59 to ‒0.22)	<.01	‒2.59	33	73.15	0.41 (0.20 to 0.80)
Secondary outcome								
Stroke/TIA	3/840	4/938	‒0.02 (‒1.46 to 1.41)	.98	‒0.03	0	0	0.98 (0.23 to 4.11)
Major bleeding	4/735	8/710	‒0.65 (‒1.80 to 0.51)	.27	‒1.10	0	0	0.52 (0.17 to 1.66)
Minor bleeding	55/840	66/938	‒0.09 (‒0.47 to 0.29)	.63	‒0.49	0	0	0.91 (0.62 to 1.33)
Total pericardial effusion	5/735	6/710	‒0.27 (‒1.47 to 0.94)	.67	‒0.43	0	0	0.77 (0.23 to 2.56)
Cardiac tamponade	2/735	3/710	‒0.36 (‒2.34 to 1.63)	.73	‒0.35	19	0.59	0.70 (0.10 to 5.11)
Total puncture complications	24/735	25/710	‒0.12 (‒0.69 to 0.44)	.68	‒0.42	0	0	0.89 (0.50 to 1.56)

Abbreviations: IA, interrupted anticoagulation group; MACCVE, major adverse cerebro‐cardiovascular events; OR, odds ratio; TIA, transient ischemic attack; UA, uninterrupted anticoagulation group.

#### 3.3.1 Primary outcomes

The UA and IA groups did not differ significantly in terms of MACCVE (log OR, ‒0.40; 95% CI, ‒1.33 to 0.53; *P *= .40; *I* ^2^, 0%) or total bleeding (log OR, ‒0.12; 95% CI, ‒0.51 to 0.27; *P *= .55; *I* ^2^, 0%). SCEs were significantly more frequent in the IA group (log OR, ‒0.90; 95% CI, ‒1.59 to ‒0.22; *P *< .01; *I* ^2^, 33%).

#### 3.3.2 Secondary outcomes

There was no significant difference in stroke or TIA incidence between the UA and IA groups (log OR, ‒0.02; 95% CI, ‒1.46 to 1.41; *P *= .98). Major and minor bleeding were also similar between the groups (*P *= .27 and *P *= .63, respectively), as were total pericardial effusion (*P *= .67), cardiac tamponade (*P *= .73), and total puncture complications (log OR, ‒0.12; 95% CI, ‒0.69 to 0.44; *P *= .68).

## 4 DISCUSSION

Catheter ablation for AF is associated with a risk for major bleeding because of multiple vascular accesses, transseptal puncture, and catheter manipulation inside left atrium.^1^, ^17^, ^18^ An international survey of AF ablation procedures found a 4.5% major complication rate.^19^ Therefore, the key pursuit is to find an optimal balance between thromboembolism and bleeding. To our knowledge, the current meta‐analysis is the first to compare procedural characteristics and embolic and bleeding events between uninterrupted and interrupted DOAC regimens for catheter ablation of AF.

### 4.1 Review of literature

The VENTURE‐AF (Study Exploring Two Treatment Strategies in Patients With Atrial Fibrillation Who Undergo Catheter Ablation Therapy) study^20^ randomized 248 patients to either uninterrupted rivaroxaban or uninterrupted warfarin. In the AXAFA‐AFNET 4 (Apixaban During Atrial Fibrillation Catheter Ablation: Comparison to Vitamin K Antagonist Therapy) study,^21^ 633 patients were randomized to uninterrupted apixaban or uninterrupted vitamin K antagonists. Neither of these studies found between‐group differences in bleeding or ischemic complication rates.^20^, ^21^ The RE‐CIRCUIT (Uninterrupted Dabigatran Etexilate in Comparison to Uninterrupted Warfarin in Pulmonary Vein Ablation) trial randomized 678 patients to either uninterrupted dabigatran or uninterrupted warfarin; those in the dabigatran arm showed a reduction in bleeding risk, with no symptomatic cerebral events.^22^ Most recently, the ELIMINATE‐AF (Edoxaban Treatment Versus Vitamin K Antagonist in Patients With Atrial Fibrillation Undergoing Catheter Ablation) trial revealed similar bleeding and ischemic complication rates for both uninterrupted edoxaban and uninterrupted warfarin.^23^

### 4.2 Heterogeneity in anticoagulation protocols

The trials described above used direct anticoagulants that have important differences in pharmacodynamics and dosing, and they also used different protocols, resulting in heterogeneity. The two studies using a once‐daily DOAC shifted the last anticoagulant dose to the night before the procedure. In VENTURE‐AF, the last dose of rivaroxaban was administered predominantly on the evening before the procedure. Patients randomized to uninterrupted edoxaban in the ELIMINATE‐AF trial also took their scheduled doses in the evening.^23^ In contrast, more than 80% of the patients treated with dabigatran in the RE‐CIRCUIT trial received the last dose < 8 hours before the ablation.^20^, ^22^ In the AXAFA‐AFNER study, apixaban treatment was continued without any dose being held back, including on the morning of the ablation.^21^

### 4.3 Guidelines

Multiple guidelines, international consensus statements, and, most recently, the European Heart Rhythm Association's *Practical Guide on the Use of Non‐Vitamin K Antagonist Oral Anticoagulants in Patients with Atrial Fibrillation* recommend continuation of oral anticoagulation with vitamin K antagonists or DOACs among patients undergoing AF ablation procedures.^5^, ^24^, ^25^ The 2017 international expert consensus statement on AF ablation supports the performing of AF ablation procedures without interruption of warfarin or DOACs (Class I), or the holding of one to two doses of the DOAC before the ablation (Class IIa).^5^ Furthermore, the European Heart Rhythm Association's Practical Guide considers it reasonable to administer a last DOAC dose 12 hours before the start of the intervention, especially when transseptal puncture will be performed without periprocedural imaging.^25^ According to the First Snapshot European Survey, truly uninterrupted antithrombotic regimens (ie, last DOAC dose shortly before the procedure) were used for approximately 30% of DOAC‐treated patients undergoing AF ablation.^6^

### 4.4 Findings from the current meta‐analysis

#### 4.4.1 Baseline characteristics

Reynolds et al^12^ studied only apixaban in two different doses, Nagao et al^13^ included apixaban, rivaroxaban, and edoxaban, and the randomized Nakamura et al^14^ and observational Nakamura et al^16^ studies included all four DOACs; Müller et al^15^ did not indicate the regimen used. In all studies, the IA group received the last DOAC dose on the day before the ablation. Bridging was done in the IA group in the studies by Müller et al and Nakamura et al^14^, ^15^ The observational study by Nakamura et al^16^ included only patients with paroxysmal AF. The randomized study by Nagao et al^13^ had a high proportion of patients with chronic kidney disease. Structural heart disease was more prevalent in the randomized studies by Reynolds et al^12^ and Nakamura et al^14^ Coronary artery disease was more prevalent in the study by Reynolds et al^12^ LVEF was relatively lower in the studies by Reynolds et al^12^ and Müller et al^15^ Protamine was used to reduce the risk for periprocedural bleeding in the Reynolds et al^12^ and Nakamura et al^14^ randomized studies, at the operator's discretion.

#### 4.4.2 Thrombosis risk

The incidence of periprocedural thromboembolism in patients with AF undergoing ablation ranges from 0.9% to 5% and depends on the diagnostic modality.^13^ Possible mechanisms include blood coming in contact with foreign surfaces, endothelial injury and inflammation in the left atrium, cellular damage and release of components, and blood flow alteration after sinus rhythm is established.^26^ Unfractionated heparin prevents common extrinsic and intrinsic coagulation pathway activation when administered before septal puncture.^14^ Artificial surface‐induced thrombosis is not prevented effectively by DOACs.^14^, ^16^, ^27^ Thus, even with UA, intraprocedural unfractionated heparin is required to prevent thromboembolic events. Moreover, there is a hypothesis that dabigatran downregulates the expression of antithrombin, with a compensatory prothrombin upregulation leading to diminution of unfractionated heparin effect.^28^

Müller et al^15^ reported greater incidence of asymptomatic, magnetic resonance imaging (MRI)‐detected, so‐called SCE in the IA group. At 1 to 2 days after radiofrequency catheter ablation, MRI was done using a 1.5 Tesla MRI scanner. Acute lesions showed focal hyperintensities in diffusion‐weighted imaging. Apparent diffusion coefficient mapping was used to differentiate true lesions from a shine‐through artifact. In the study by Nagao et al,^13^ SCE was independently predicted by CHA_2_DS_2_‐VASc score in the UA group and by intraprocedural cardioversion and procedure time in the IA group. Overall, SCE was significantly more frequent in the IA group (*P *< .005).^13^ The observational study by Nakamura et al^16^ found that interrupted dabigatran was an independent predictor of SCE. The SCE rate did not differ significantly between the UA and IA groups in the randomized study by Nakamura et al^14^ In our meta‐analysis, the incidence of groin complications or tamponade did not differ significantly between the UA and IA groups, but SCE was significantly more frequent with IA, further emphasizing the utility of UA regime before ablation. This is supported by the need for a higher total heparin dose in the IA group. Moreover, ACT before first heparin bolus was significantly longer in the UA cohort, supporting lesser thrombotic risk in this group.

#### 4.4.3 Bleeding risk

Reynolds et al^12^ stated that patients taking DOACs may have lower risk for periprocedural bleeding than patients taking warfarin. The randomized trial by Nakamura et al^14^ found similar rebleeding rates at venous puncture sites in both the UA and IA groups. Although the presence of chronic kidney disease increased periprocedural bleeding risk in a study by Yanagisawa et al,^29^ similar findings were not reported in the studies incorporated in this meta‐analysis. The same study found antiplatelet use to be an independent predictor of adverse events in AF ablation; conversely, Reynolds et al^12^ reported that aspirin was not significantly associated with bleeding in multivariate model results.^29^ Several studies found low rates of major bleeding in both UA and IA groups and similar incidences of minor bleeding, which was attributed to postprocedural protamine use and postprocedural unfractionated heparin use.^12^, ^13^, ^14^ In keeping with the above findings, total bleeding, major bleeding, and minor bleeding were similar in the two groups in our meta‐analysis. Similarly, total pericardial effusion, cardiac tamponade, and total puncture complications did not differ significantly between the IA and UA groups, nor did protamine use. A recently published meta‐analysis found that the rate of vascular complications in electrophysiology procedures—and thus, major and minor bleeding—can be reduced by using ultrasound‐guided femoral access.^30^

#### 4.4.4 MACCVE

MACCVE is a novel composite endpoint, we looked into, which comprised of major bleeding events as well as thrombotic events. In our meta‐analysis, MACCVE did not differ significantly between the UA and IA groups. Although SCE was noted more in relation to interrupted DOACs, the overall outcomes were comparable between the two groups which suggest that even with uninterrupted periprocedural anticoagulation, patients can be discharged safely from hospital following AF ablation on the same day.^29^

### 4.5 Predictors of silent cerebral events

To date, the clinical relevance of SCE remains unclear. Some data suggest that SCE is associated with cognitive impairment occurring after an AF ablation procedure.^31^ This represents a real cause for concern for some authors,^21^, ^32^ whereas the relationship between SCE and cognitive impairment is disputed by others.^2^, ^7^, ^33^ Increased incidence of SCE has been reported with reinsertion and application of a previously withdrawn cryoballoon, multielectrode catheter use for additional left atrial mapping, and transient coronary air embolism.^34^ Additional radiofrequency ablation within the left atrium in patients undergoing nonpulmonary vein isolation ablation was an independent risk factor for cerebral ischemic events in a study by Nakamura et al^35^ In a very recent meta‐analysis published, uninterrupted DOAC was found to have similar bleeding events with comparison to minimally interrupted DOAC and also mirrored our findings of lesser SCE.^36^ However, this study did not explore the procedural aspects, especially in relation to use of heparin and ACT. Also our results are statistically more relevant as we accounted the necessary modifications to address sparse binary events.

## 5 LIMITATIONS

First, we were able to include only five studies, two of which were observational trials. Second, the overall follow‐up duration was less. Third, there was considerable difference in the periprocedural anticoagulation regime across the studies. Fourth, subgroup analyses (eg, paroxysmal vs persistent AF, mapping vs balloon strategy) could not be done because of lack of data since the data are heterogeneous and in consequence subgroups are small. Moreover, two of the five studies did not report kidney function (GFR), while this is relevant especially with DOACS. The presentation of atrial fibrillation differed significantly between the studies (paroxysmal in one 100%, others only, or less than 50%), which implies that the patients were affected by persistent or permanent AF. This may have an impact on rhythm variability, which may play a role in developing silent cerebral ischemia. The observational study by Nakamura^16^ differed from the others for some specific features, the most relevant of which is that it was specifically focused on the very perioperative period, and especially on SCEs; the limitation of the follow‐up at the first day after ablation (up to discharge, for clinical events) made this study significantly different from the other four. Moreover, patients with severe bleedings were intentionally excluded from the study, although their number turned out to be negligible. Then, despite being true that patients were on all the four DOACS, as reported, the study protocol requested the shift to dabigatran be done for all patients on the very day of the procedure, till the next day. Finally, given the infrequent outcomes, the overall sample size (despite pooling the number of patients) across the studies may be inadequate.

## 6 CONCLUSION

Compared with interrupted DOAC therapy, uninterrupted DOACs during AF ablation were associated with similar bleeding events and similar procedural times but lower rates of SCE, despite achieving a similar mean ACT. Further research is needed for risk stratification of the various DOAC regimens, understanding the predictors of SCE, and long‐term follow‐up of patients with SCE. On the basis of the information available thus far, we recommend truly uninterrupted DOAC treatment at the time of AF ablation.

## 7 DECLARATION

- Ethics approval and consent to participate‐ Not applicable
- Consent for publication‐ not applicable
- Competing interests‐ none
- Funding‐ none
- Author contributions‐ IBR, DK, AM, PK, SKS, AA,JB, JJ, and AK
  - Conception and design: IBR, DK, PK, AK, and ST
  - Analysis and interpretation of data: IBR, DK, AM, PK, SKS, and ST
  - Drafting of the manuscript or revising it critically for important intellectual content: IBR, DK, AM, PK, SKS, AA, JB, JJ, and AK
  - Final approval of the manuscript: IBR, DK, AM, PK, SKS, AA, JB, JJ, and AK
- Acknowledgments: Jeanie F. Woodruff, BS, ELS, contributed to the editing of this manuscript
- Supporting data are available and can be accessed from the corresponding author
- **Disclosure statement**‐ No conflict of interest to be declared

## Supporting information

Supplementary Material
