# Supplementary material for: Outcomes of uninterrupted vs interrupted Periprocedural direct oral Anticoagulants in atrial Fibrillation ablation: A meta‐analysis
Source: J Arrhythm. 2021 Jan 29;37(2):384–93. doi: 10.1002/joa3.12507 (PMC8021981; doi:10.1002/joa3.12507)
Supplement: Supplementary file 1 — Supplementary Material [file JOA3-37-384-s001.docx]

# Online Supplement

#### eTable 1. Quality Assessment of the Selected Studies

| Risk of Bias 2.0 Scale | Randomized Studies | | | |
| --- | --- | --- | --- | --- |
| Points | **Reynolds et al. 2018^1^** | **Nagao et al. 2019^2^** | | **Nakamura et al. 2019^3^** |
| Random sequence generation | – | + | | + |
| Allocation concealment | + | + | | + |
| Blinding of participants and personnel | + | + | | + |
| Blinding of outcome assessment | + | + | | + |
| Incomplete outcome data | + | + | | + |
| Selective reporting | + | + | | + |
| Other bias | + | + | | + |
| Newcastle-Ottawa scale (case-control) | **Observational Studies** | | | |
| Points | **Müller et al. 2016^4^** | | **Nakamura et al. 2019^5^** | |
| Selection | **** | | *** | |
| Comparability | * | | * | |
| Outcome | * | | ** | |
| Total score | 6 | | 7 | |

#### eTable 2. Clinical Outcomes Across the Selected Studies

|  | Randomized Studies | | | | | | Observational Studies | | | | |
| --- | --- | --- | --- | --- | --- | --- | --- | --- | --- | --- | --- |
|  | **Reynolds et al. 2018^1^** | | **Nagao et al. 2019^2^** | | **Nakamura et al. 2019^3^** | | | **Müller et al. 2016^4^** | | **Nakamura et al. 2019^5^** | |
|  | **UA (n=150)** | **IA (n=145)** | **UA (n=100)** | **IA (n=100)** | **UA (n=421)** | **IA (n=423)** | | **UA (n=64)** | **IA (n=42)** | **UA (n=105)** | **IA (n=228)** |
| Primary outcomes |  |  |  |  |  |  | |  |  |  |  |
| MACCVE | 4 | 5 | 0 | 3 | 4 | 6 | | 0 | 0 | 1 | 1 |
| Total bleeding | 17 | 18 | 6 | 9 | 27 | 27 | | 4 | 4 | – | – |
| Secondary outcomes |  |  |  |  |  |  | |  |  |  |  |
| Silent stroke | – | – | 4 | 17 | 69/348 | 69/313 | | 8 | 15 | 14 | 68 |
| Cerebral embolic stroke | 1 | 1 | 0 | 1 | 1 | 1 | | 0 | 0 | 1 | 1 |
| TIA | 1 | 1 | 0 | 1 | 1 | 1 | | 0 | 0 | 0 | 0 |
| Systemic embolism | 0 | 0 | 0 | 0 | 0 | 0 | | 0 | 0 | 0 | 0 |
| Major bleeding | 2 | 3 | 0 | 1 | 2 | 4 | | 0 | 0 | – | – |
| Cardiac tamponade | 2 | 0 | 0 | 1 | 0 | 2 | | 0 | 0 | – | – |
| Pseudoaneurysm | 0 | 1 | 1 | 1 | 1 | 0 | | 0 | 0 | – | – |
| RPH | 0 | 0 | 0 | 0 | 1 | 0 | | 0 | 0 | – | – |
| ICH | 0 | 0 | 0 | 0 | 0 | 2 | | 0 | 0 | – | – |
| Minor bleeding | 15 | 15 | 6 | 8 | 25 | 23 | | 4 | 4 | 5 | 16 |
| Groin hematoma | 0 | 3 | 4 | 6 | 7 | 8 | | 0 | 0 | 4 | 9 |
| Minor pericardial effusion | 0 | 0 | 0 | 0 | 3 | 3 | | 0 | 0 | 1 | 6 |
| Rebleeding from venous sites | – | – | – | – | 10 | 10 | | – | – | – | – |
| Other bleeding | – | – | – | – | 5 | 2 | | – | – | – | – |
| Other composites |  |  |  |  |  |  | |  |  |  |  |
| Total pericardial effusion | 2 | 0 | 0 | 1 | 3 | 5 | | 0 | 0 | – | – |
| Total puncture complications | 0 | 4 | 5 | 7 | 19 | 18 | | 0 | 0 | – | – |

Abbreviations: IA, interrupted anticoagulation group; ICH, intracranial hemorrhage; MACCVE, major adverse cerebrocardiovascular events; RPH, retroperitoneal hematoma; TIA, transient ischemic attack; UA, uninterrupted anticoagulation group.

#### eFigure 1. Flow Diagram of the Literature Search


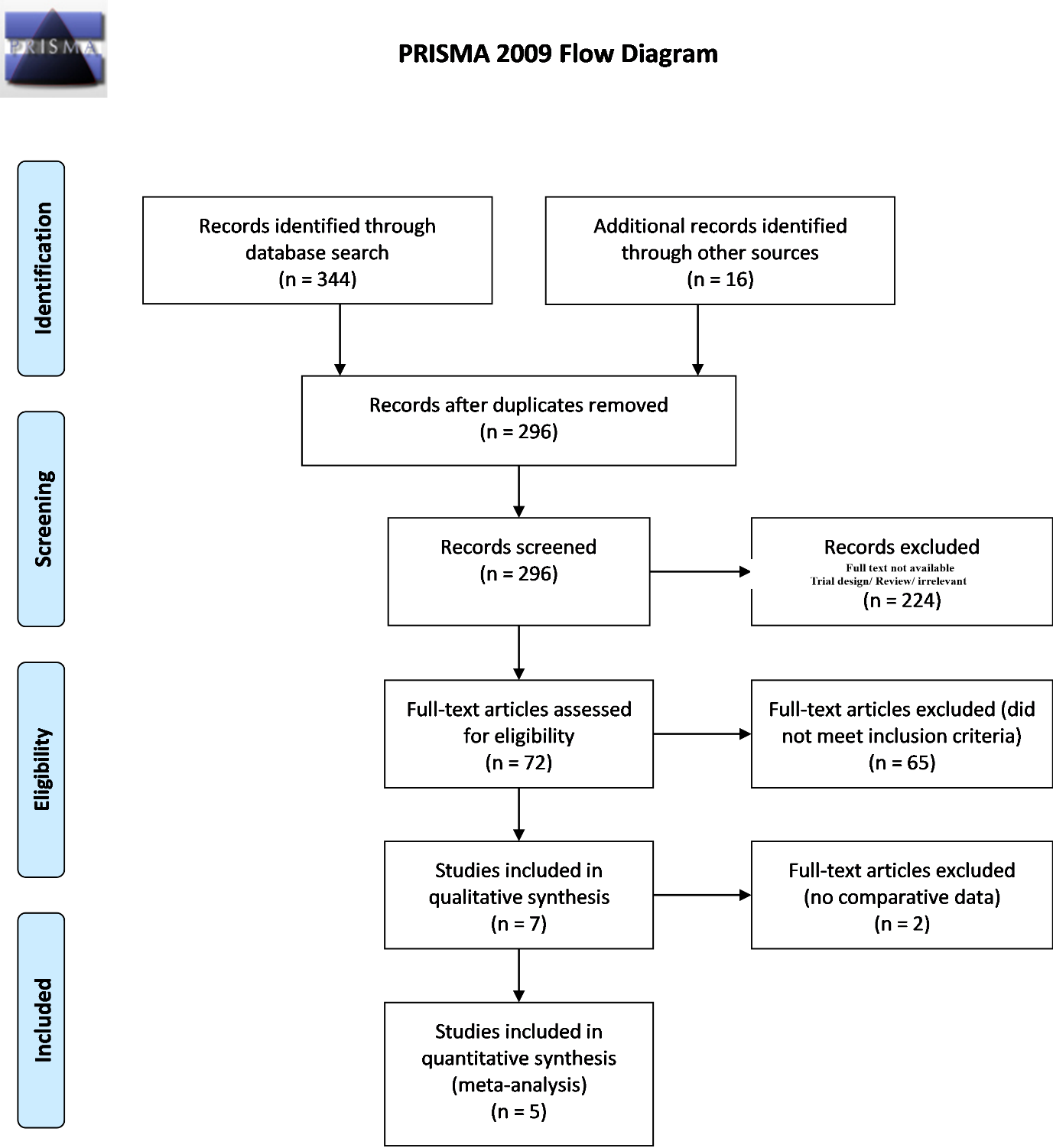


####
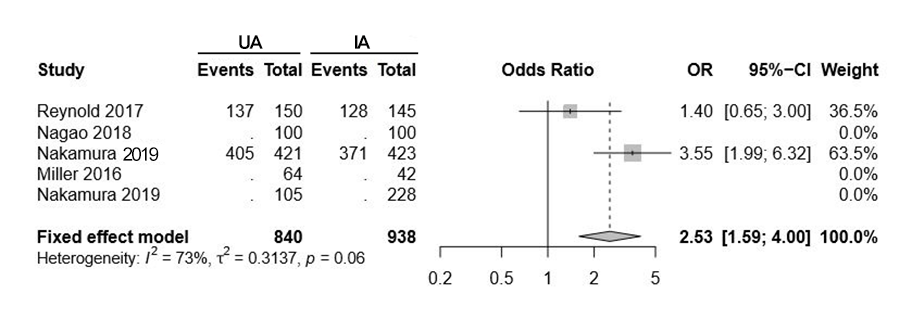
eFigure 2. Comparison of Protamine Use between Two Groups

Abbreviations: IA, interrupted anticoagulation group; OR, odds ratio; UA, uninterrupted anticoagulation group.

**References**

1. Reynolds MR, Allison JS, Natale A, et al. A prospective randomized trial of apixaban dosing during atrial fibrillation ablation: the AEIOU Trial. *JACC Clin Electrophysiol.* 2018;4(5):580-88.

2. Nagao T, Suzuki H, Matsunaga S, et al. Impact of periprocedural anticoagulation therapy on the incidence of silent stroke after atrial fibrillation ablation in patients receiving direct oral anticoagulants: uninterrupted vs. interrupted by one dose strategy. *Europace.* 2019;21(4):590-97.

3. Nakamura K, Naito S, Sasaki T, et al. Uninterrupted vs. interrupted periprocedural direct oral anticoagulants for catheter ablation of atrial fibrillation: a prospective randomized single-centre study on post-ablation thrombo-embolic and haemorrhagic events. *Europace.* 2019;21(2):259-67.

4. Müller P, Halbfass P, Szöllösi A, et al. Impact of periprocedural anticoagulation strategy on the incidence of new-onset silent cerebral events after radiofrequency catheter ablation of atrial fibrillation. *J Interv Card Electrophysiol.* 2016;46(3):203-11.

5. Nakamura R, Okishige K, Shigeta T, et al. Clinical comparative study regarding interrupted and uninterrupted dabigatran therapy during perioperative periods of cryoballoon ablation for paroxysmal atrial fibrillation. *J Cardiol.* 2019;74(2):150-55.
